# *Raet1e* Polymorphisms Are Associated with Increased Risk of Developing Premature Coronary Artery Disease and with Some Cardiometabolic Parameters: The GEA Mexican Study

**DOI:** 10.1155/2018/1847696

**Published:** 2018-12-18

**Authors:** Rosalinda Posadas-Sánchez, Bladimir Roque-Ramírez, José Manuel Rodríguez-Pérez, Nonanzit Pérez-Hernández, José Manuel Fragoso, Teresa Villarreal-Molina, Ramón Coral-Vázquez, Maria Elizabeth Tejero-Barrera, Carlos Posadas-Romero, Gilberto Vargas-Alarcón

**Affiliations:** ^1^Departamento de Endocrinología, Instituto Nacional de Cardiología Ignacio Chávez, Ciudad de México, Mexico; ^2^Laboratorio de Nutrigenética y Nutrigenómica, Instituto Nacional de Medicina Genómica (INMEGEN), Ciudad de México, Mexico; ^3^Departamento de Biología Molecular, Instituto Nacional de Cardiología Ignacio Chávez, Ciudad de México, Mexico; ^4^Laboratorio de Genómica Cardiovascular, Instituto Nacional de Medicina Genómica (INMEGEN), Ciudad de México, Mexico; ^5^Sección de Estudios de Posgrado e Investigación, Escuela Superior de Medicina, Instituto Politécnico Nacional, Ciudad de México, Mexico

## Abstract

In an animal model, new evidence has been reported supporting the role of *raet1e* as an atherosclerosis-associated gene. Our objective was to establish if *raet1e* polymorphisms are associated with the risk of developing premature coronary artery disease (CAD) or with the presence of cardiometabolic parameters. After an informatic analysis, five polymorphisms were chosen and determined in 1158 patients with premature CAD and 1104 controls using 5′ exonuclease TaqMan genotyping assays. Standardized questionnaires were applied to all participants to obtain family medical history, demographic information, history of nutritional habits, physical activity, alcohol consumption, and pharmacological treatment. The functional effect of the rs7756850 polymorphism was analyzed by luciferase assays. Under different models, adjusted by age, gender, body mass index, current smoking, and type 2 diabetes mellitus, the rs6925151 (OR = 1.250, *p*_heterozygote_ = 0.026; OR = 1.268, *p*_codominant1_ = 0.034), rs9371533 (OR = 1.255, *p*_heterozygote_ = 0.024), rs7756850 (OR = 1.274, *p*_heterozygote_ = 0.016; OR = 1.294, *p*_codominant1_ = 0.031), and rs9383921 (OR = 1.232, *p*_heterozygote_ = 0.037) polymorphisms were associated with increased risk of premature CAD. When compared to the rs7756850 *G* allele, the *C* allele showed a decreased luciferase activity. In premature CAD patients, associations with low levels of adiponectin, with a high presence of hypertension, and with high levels of gamma-glutamyltransferase and total cholesterol were observed. In healthy controls, associations with a decrease in LDL pattern B, aspartate aminotransaminase, and hypo-*α*-lipoproteinemia were detected. An association of the *raet1e* polymorphisms with an increased risk of developing premature CAD and with cardiometabolic parameters has been shown for the first time. In addition, the functional effect of the rs7756850 polymorphism was defined.

## 1. Introduction

Acute coronary artery disease (CAD) is the most common cause of death in industrialized countries, accounting for up to 40% of all deaths [1]. CAD is a multifactorial pathology resulting from an atherosclerotic process that develops and progresses for decades prior to an acute event. Atherosclerosis is considered a low-grade inflammatory state of the intima (inner lining) of medium- and large-sized arteries; this inflammatory state is accelerated by the well-known classical risk factors, such as smoking, diabetes, obesity, high blood pressure, high cholesterol, and genetics [2]. In the last decades, significant advances have been made in identifying chromosomal loci linked to genetic variations that confer susceptibility to CAD; both candidate-gene and genome-wide association studies (GWAS) have been part of the strategies used [3–5]. Important contributions have been made using animal models, which have allowed for the detection of atherogenic loci. Intercrosses and backcrosses between strains varying in complex disease-related traits have been performed, and quantitative trait locus (QTL) mapping has been used to identify the modifier loci [6]. Using this strategy, a region called *Ath* locus has been identified [7]. This is a complex locus with a 10a proximal region in females containing 21 genes and a 10b distal region in both sexes containing 7 genes (*Pde7b*, *Ahi1*, *Myb*, *Hbs1L*, *Aldh8a1*, *Sgk1*, and *Raet1e*) [8]. Rodríguez et al., using a sequence analysis of these genes in a subcongenic mouse line, revealed *raet1e* (encodes Raet1, a major histocompatibility complex class 1-like molecule expressed in lesion aortic endothelial cells and macrophage-rich regions) as a novel atherosclerosis gene [9]. Raet1 is a family of molecules that are ligands of the NKG2D receptor present in NK and in a variety of T cell subsets, including CD8^+^ cytotoxic T cells [10]. The NKG2D system itself in an activated state also releases pro- and anti-inflammatory cytokine transcripts to establish either communication with other cells or self-regulation. The role of raet1 in NKG2D system activation and, in consequence, in the production of pro- and anti-inflammatory cytokines could be the link between the molecule-encoding gene and the development of atherosclerosis. Accordingly, our objective was to analyze the possible association of the *raet1e* polymorphisms and CAD development in a cohort of Mexican patients. After an informatics analysis, we included 5 polymorphisms in our study, which were selected for their possible functional effects and/or their informative nature (minor allele frequency > 5%).

## 2. Material and Methods

### 2.1. Subjects

The present study included 2262 individuals (1158 patients with premature CAD and 1104 healthy controls) belonging to the Genetics of Atherosclerotic Mexican Study (GEA). Premature CAD was defined as history of myocardial infarction, angioplasty, revascularization surgery, or coronary stenosis > 50% on angiography, diagnosed before age 55 in men and before age 65 in women. Controls were apparently healthy asymptomatic individuals without family history of premature CAD, recruited from blood bank donors and through brochures posted in social service centers. Standardized questionnaires were applied to all participants to obtain family medical history, demographic information, history of nutritional habits, physical activity, alcohol consumption, and pharmacological treatment.

Ancestry estimation of premature CAD patients and healthy controls was determined and reported previously [11]. Mean global ancestry was not significantly different between patients and controls (55.8% vs 54.0% Amerindian ancestry, 34.3% vs 35.8% Caucasian, and 9.8% vs 10.1% African mean ancestry for patients and controls, respectively, *p* > 0.05), strongly suggesting that population stratification was not a bias or confounding factor in this study.

The GEA study was approved by the Bioethics Committee of the Instituto Nacional de Cardiología Ignacio Chávez (INCICH) and aligned to the 1975 Declaration of Helsinki. All participants provided informed consent.

### 2.2. Biochemical and Clinical Characteristics

Biochemical, anthropometric, metabolic, and cardiovascular risk factors were evaluated in both premature CAD cases and controls and defined as previously described [12–14]. Briefly, after a 12 h fast, blood samples were collected from the participants. Plasma triglycerides, total and high-density lipoprotein (HDL) cholesterol, and serum aspartate aminotransferase (AST) and gamma glutamyltransferase (GGT) were measured in fresh samples, using standardized enzymatic procedures in a Hitachi 902 analyzed (Hitachi Ltd, Tokyo, Japan). LDL-C was estimated using the DeLong et al. formula [15]. Enzyme-linked immunosorbent assay (ELISA) technique (R&D Systems Quantikine Kit, Minneapolis, Minnesota, USA) was used to measured total serum adiponectin, the intra- and interassay variation coefficients were < 10%.

Height, weight, and waist circumference were measured, and BMI was calculated as weight in kilograms divided by height in meters squared. After subjects rested for at least 10 minutes, systolic and diastolic blood pressures were measured, and the average of the second and third measurements was used as the blood pressure of the subject. Hypercholesterolemia was defined as total cholesterol (TC) levels > 200 mg/dL. Hypoalphalipoproteinemia was defined as HDL − cholesterol < 50 mg/dL and < 40 mg/dL in women and men, respectively. Type 2 diabetes mellitus was defined by the American Diabetes Association criteria [16] and was also considered when participants reported glucose lowering treatment or a physician diagnosis of diabetes. The current use of antihypertensive medication as well as systolic blood pressure ≥ 140 mmHg or diastolic blood pressure ≥ 90 mmHg were used to defined hypertension. LDL particle size was estimated with the LDL-cholesterol/apoB ratio, and LDL pattern B was considered with a LDL-cholesterol/apoB ratio ≤ 1.2, which corresponded to an LDL diameter of 25.5 nm [17]. Low adiponectin was defined as serum adiponectin levels ≤ 25th percentile (≤8.67 *μ*g/mL in women and ≤5.30 *μ*g/mL in men). Elevated aspartate aminotransaminase (AST) and gamma glutamyltransferase (GGT) were defined as AST activity (25 IU/L in women and 28 IU/L in men) and GGT activity (28 IU/L in women and 34 IU/L in men) ≥75th percentile. These cutoff points were obtained from a GEA study sample of 101 men and 180 women without obesity and with normal values of blood pressure and fasting glucose and lipids.

### 2.3. Computed Axial Tomography Study

Computed tomography of the chest and abdomen was performed using a 64-channel multidetector helical computed tomography system (Somatom Sensation, Siemens) and interpreted by experienced radiologists. Scans were read to assess and quantify the CAC score using the Agatston method [18]; total, subcutaneous, and visceral abdominal fat areas (TAF, SAF, and VAF) as described by Kvist et al. [19] and the hepatic to splenic attenuation ratio as described by Longo et al. [20].

### 2.4. Genetic Analysis

The 5′ exonuclease TaqMan genotyping assays were used to determine the *raet1e* (rs6925151, rs9371533, rs7756850, rs2151910, and rs9383921) polymorphisms. The determinations were made with an ABI Prism 7900HT Fast Real-Time PCR system, according to manufacturer's instructions (Applied Biosystems, Foster City, CA, USA). Previously sequenced samples of the different genotypes were included as positive controls.

### 2.5. Functional Prediction Analysis

In order to predict the potential effect of the *raet1e* polymorphisms, we used the following bioinformatics tools: SNP Function Prediction [21], Human-transcriptome Database for Alternative Splicing [22], SplicePort: An Interactive Splice Site Analysis Tool [23], SNPs3D [24], PESX: Putative Exonic Splicing enhancers/Silencers [25], and ESEfinder release 3.0 [26].

### 2.6. Constructs and Plasmids

**Figure 1:**
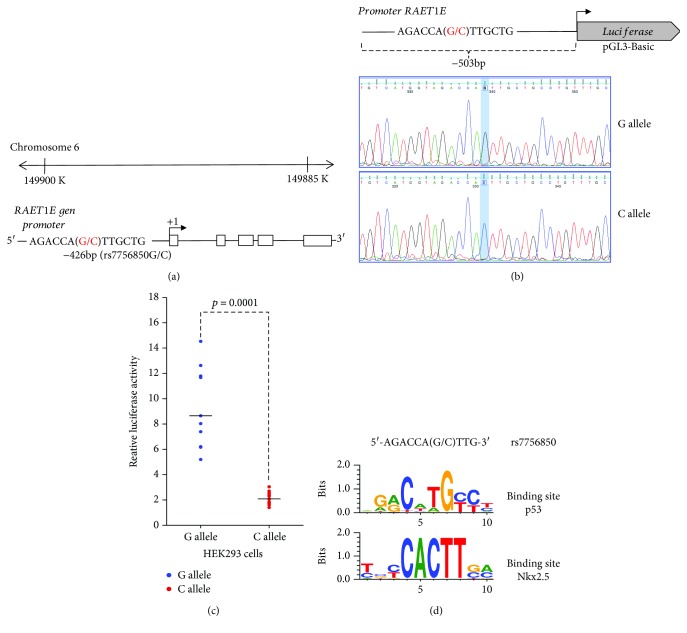
rs7756850 affects transcriptional activity of *raet1e* promoter sequence. (a) Schematic diagram of the *raet1e* gene and location of allelic variants on promoter sequence (−426 bp, *G/C*). (b) Construction of plasmid. The promoter sequence with each variant was cloned in the pGL3 vector into *Mlu*I*/Xho*I sites to direct the expression of the luciferase gene. Both constructions (*G* allele and *C* allele) were confirmed by sequencing. (c) Dual-luciferase assay in HEK293 cells. The presence of the *C* allele reduces the promoter activity of the cloned sequence (−503 bp) in ≈85% as compared with the promoter activity of the sequence harboring the *G* allele (*p* = 0.0001). Lines represent the median of four independent experiments performed in triplicate (*n* = 11). *p* < 0.05 was determined by the Mann-Whitney *U* test. (d) *In silico* analysis suggests that variant rs7756850 modifies the consensus site for p53 and Nkx2.5 transcription factors. Due to the *raet1e* gene orientation into chromosome 6, the *G* allele is considered the ancestral allele in the luciferase assays.

A 503 bp DNA fragment of the *raet1e* regulatory sequence (−460/+43) was amplified by PCR from genomic DNA of homozygous individuals for the rs7756850 variant (Figure 1(a)) and cloned upstream of the luciferase reporter gene into *Mlu*I/*Xho*I sites of the pGL3-Basic vector (Promega) to generate the constructions *pRAET1EgLUC* (*G* allele) and *pRAET1EcLUC* (*C* allele) (Figure 1(b)). Primers used to amplify this sequence were forward 5′-GCGCACGCGTCACACACACAAAACCCATCTG-3′ and reverse 5′-GCGCCTCGAGGGCACTGCCCAAATTCTTTA-3′. Polymerase chain reaction conditions were as follows: 1 cycle at 95°C for 3 minutes; 33 cycles at 95°C for 30 s, 62°C for 30 s, and 72°C for 30 s; and a final extension at 72°C for 5 minutes. Integrity of the clone was confirmed by DNA sequencing (Figure 1(b)).

### 2.7. Cell Culture and Transfection

HEK293 cells were grown in Dulbecco's modified Eagle's medium (DMEM) (Invitrogen Life Technologies Inc., Carlsbad, CA, USA) supplemented with 10% fetal bovine serum and 1% antibiotics-antimycotics (Invitrogen) at 37°C with 5% CO_2_. A total of 600,000 cells were seeded in six-well plates in DMEM supplemented with 4% fetal bovine serum (Invitrogen). Twenty-four hours later, the cells were transiently transfected using Lipofectamine 3000 (Invitrogen) with 2 *μ*g of each promoter construct. The Plos pGL3-basic vector without insert was used as a negative control. For all assays, 300 ng of pRL/CMV *Renilla reniformis* luciferase vector (Promega) was cotransfected for normalization.

### 2.8. Dual-Luciferase Assay

HEK293 cells were harvested forty-eight hours post-transfection. Luciferase activity was measured using the Dual Luciferase Reporter Assay System (Promega) according to the manufacturer's instructions with a TD-20/20 luminometer (Turner BioSystems, Sunnyvale, CA).

### 2.9. Statistical Analysis

The analysis was made using the SPSS version 15.0 statistical package (SPSS, Chicago, Il). Means, medians, interquartile ranges, and frequencies were calculated as the case may be. Continuous and categorical variables were analyzed by Student's *T* test, Mann-Whitney *U* test, Kruskal-Wallis, and chi-square or Fisher test as appropriate. We analyzed the polymorphism associations with premature CAD and other variables using logistic regression under the following inheritance models: additive, codominant 1, codominant 2, dominant, heterozygote, and recessive. The models were adjusted by age, gender, body mass index, current smoking, and type 2 diabetes mellitus. Statistical power to detect the association of the polymorphisms with pCAD was estimated with QUANTO software (http://biostats.usc.edu/Quanto.html). For rs6925151, polymorphism was 75.2% (heterozygote model) and 80.3% (codominant 1 model), for rs9371533 it was 76.9% (heterozygote model), for rs7756850 it was 81.9 (heterozygote model) and 86% (codominant 1 model), and for rs9383921 it was 69.7% (heterozygote model). Logistic regression analyses were performed to assess the associations of *raet1e* polymorphisms with metabolic parameters and cardiovascular risk factors under different inheritance models and adjusting for age, gender, and BMI, as appropriate. Genotype frequencies did not deviate from the Hardy-Weinberg equilibrium in any case (HWE, *p* > 0.05).

## 3. Results

### 3.1. Biochemical, Clinical, Demographic, Lifestyle, and Tomographic Characteristics

The GEA study originally included 1500 healthy controls; yet, after the computed tomography, the CAC score of 396 subjects was greater than zero. They were thus considered as individuals with subclinical atherosclerosis and were not included in the analysis. The final control group included 1104 individuals with a CAC score of zero. The CAC was not measured in pCAD patients because the presence of stents and previous coronary surgery results in artifacts that do not allow the correct interpretation of tomographic images; thus, the CAC score of pCAD patients was not reported.

Several biochemical, clinical, demographic, lifestyle, and tomography differences were observed between premature CAD patients and healthy controls (Table 1). As expected, some characteristics such as total cholesterol (mg/dL), low-density lipoprotein-cholesterol (mg/dL), total cholesterol > 200 mg/dL, and smoking habit were higher in the control group than in premature CAD patients. This result is due to the patients who are diagnosed receive statin treatment in addition to their lifestyle change. As can be seen in the table, systolic and diastolic blood pressure in patients shows statistical difference compared to the control group, but the range of this values is within normal values; nevertheless, 68% of the CAD group are presented as hypertensive. The reason for this discrepancy is that some patients with hypertension are under treatment and in consequence their pressure levels were within the normal range.

### 3.2. Association of the *raet1e* Polymorphisms with Premature CAD

The association of the *raet1e* polymorphisms with CAD is shown in Table 2. Under different models adjusted by age, gender, body mass index, current smoking, and type 2 diabetes mellitus (T2DM), rs6925151 (OR = 1.250, 95% CI: 1.027-1.521, *p*_heterozygote_ = 0.026; OR = 1.268, 95% CI: 1.018-1.581, *p*_codominant1_ = 0.034), rs9371533 (OR = 1.255, 95% CI: 1.031-1.527, *p*_heterozygote_ = 0.024), rs7756850 (OR = 1.274, 95% CI: 1.047-1.550, *p*_heterozygote_ = 0.016; OR = 1.294, 95% CI: 1.024-1.635, *p*_codominant1_ = 0.031), and rs9383921 (OR = 1.232, 95% CI: 1.012-1.499, *p*_heterozygote_ = 0.037) were all associated with an increased risk of developing CAD.

### 3.3. Association of the *raet1e* Polymorphisms with Metabolic and Clinical Parameters

*Raet1e* polymorphism associations with clinical and metabolic parameters were analyzed separately in premature CAD patients and healthy controls (Tables 3 and 4, respectively). The associations found in premature CAD patients under different models adjusted by age, gender, and BMI were as follows (Table 3). rs9371533 was associated with a raised risk of hypertension (OR = 1.264, 95% CI: 1.054-1.515, *p*_add_ = 0.012; OR = 1.501, 95% CI: 1.092-2.063, *p*_rec_ = 0.012; OR = 1.629, 95% CI: 1.125-2.358, *p*_cod2_ = 0.010). An association of rs9371533 (OR = 1.437, 95% CI: 1.071-1.926, *p*_het_ = 0.016) and rs2151910 (OR = 1.515, 95% CI: 1.133-2.027, *p*_het_ = 0.005; OR = 1.508, 95% CI: 1.108-2.053, *p*_cod1_ = 0.009) with an augmented risk of having total cholesterol levels > 200 mg/dL was found. Both rs7756850 (OR = 1.436, 95% CI: 1.080-1.909, *p*_cod1_ = 0.013) and rs9383921 (OR = 1.432, 95% CI: 1.078-1.903, *p*_cod1_ = 0.013) showed a significant association with raised levels of GGT, whereas rs7756850 (OR = 1.237, 95% CI: 1.042-1.467, *p*_add_ = 0.015; OR = 1.493, 95% CI: 1.119-1.991, *p*_rec_ = 0.006; OR = 1.538, 95% CI: 1.096-2.185, *p*_cod2_ = 0.013) and rs9383921 (OR = 1.257, 95% CI: 1.059-1.490, *p*_add_ = 0.009; OR = 1.509, 95% CI: 1.1129-2.016, *p*_rec_ = 0.005; OR = 1.600, 95% CI: 1.132-2.260, *p*_cod2_ = 0.008) were all associated with low levels of adiponectin.

In healthy controls (Table 4), moreover, the following associations were found under different models adjusted by age, gender, and BMI. An association of rs9371533 (OR = 0.694, 95% CI: 0.545-0.885, *p*_het_ = 0.003; OR = 0.658, 95% CI: 0.490-0.884, *p*_cod1_ = 0.005) with a decreased risk of hypo-*α*-lipoproteinemia was found. rs566850 (OR = 0.815, 95% CI: 0.690-0.964, *p*_add_ = 0.017) and rs9383921 (OR = 0.710, 95% CI: 0.533-0.946, *p*_rec_ = 0.019) were nominally associated with a decreased risk of having LDL pattern B. Finally, both rs7756850 (OR = 0.720, 95% CI: 0.549-0.944, *p*_dom_ = 0.018) and rs93833921 (OR = 0.800, 95% CI: 0.673-0.951, *p*_add_ = 0.011; OR = 0.695, 95% CI: 0.695-0.911, *p*_dom_ = 0.008; OR = 0.651, 95% CI: 0.461-0.919, *p*_cod2_ = 0.015) were associated with a diminished risk of AST > p75.

### 3.4. Luciferase Assays

Because the in silico analysis suggests that rs7756850 modifies important binding sites for transcription factors, we decided to examine the effect of either allele *G* or *C* on the promoter activity. To this end, we transfected HEK293 cells with a luciferase reporter gene construct harboring 503 bp of the *raet1e* regulatory sequence with the *G* or *C* allele of the rs7756850 polymorphism. Interestingly, cells transfected with the construct *pRAET1EcLUC* (*C* allele) showed an ≈85% reduction in luciferase activity (*p* = 0.0001) compared with those transfected with *pRAET1EgLUC* (*G* allele) (Figure 1(c)).

## 4. Discussion

*Reat1e* is a new gene recently associated with the development of atherosclerosis in animal models [9]. The role of this gene product as a ligand of the NKG2D receptor (present in several T cell subsets) and its subsequent effect in the release of pro- and anti-inflammatory cytokines support this association. In the present work, we analyzed the distribution of 5 polymorphisms of the *raet1e* gene in both CAD patients and healthy controls; our goal was to establish the *raet1e* gene's role in the susceptibility of developing this disease and its association with cardiometabolic parameters. While four out of five polymorphisms were associated with an increased risk of developing premature CAD, some of them were associated with several metabolic and clinical parameters in both patients and controls. In premature CAD patients, associations with low levels of adiponectin, high levels of GGT and TC, and a high presence of hypertension were observed. In healthy controls, on the other hand, associations with a decrease in LDL pattern B, AST, and hypo-*α*-lipoproteinemia were detected. As can be seen, the associations with metabolic and clinical parameters were different in patients and healthy controls.

Of note, there was a lack of previous studies on the *raet1e* polymorphisms with which to compare our results. Nevertheless, in the same cohort of patients and healthy controls, other polymorphisms in candidate genes have been associated with both premature CAD and cardiometabolic parameters [11, 27–29]. The five polymorphisms included in the present study are functional and informative (frequency of the minor allele > 10%) according to an informatics analysis. The rs7756850 polymorphism is located in the promoter region and produces a binding site for the Nkx-2 cardiac transcription factor, which regulates tissue-specific gene expression involved in early heart formation and development. Mutations in the NKx-2-encoding gene can cause different forms of congenital heart defects, including ventricular and atrial septal defect, atrial ventricular block, and Tetralogy of Fallot [30]. In a recent study, Kontaraki et al. [31] analyzed the expression levels of some early cardiac marker genes, including Nkx-2, in peripheral blood mononuclear cells of patients with CAD and healthy individuals. When compared to healthy controls, they demonstrated a higher expression of these markers in CAD patients; in the same way, they found higher transcript levels in patients with a more severe disease. Herein, the functional effect of rs7756850 was demonstrated using a luciferase assay. Luciferase activity assay shows that the rs7756850 variant modifies the transcriptional activity of the *raet1e* promoter in ≈85% in HEK293 cells (Figure 1(c)). The change in luciferase activity may be due to the multiple binding sites that are affected by this variant (Figure 1(d)). The *G* allele preserves the binding site to transcription factor p53 (NGRCWTGYYY); this transcription factor is of ubiquitous expression and positively modulates multiple genes [32]. On the other hand, the *C* allele eliminates the binding site p53 and consequently suggests a negative modulation of the *raet1e* promoter (Figure 1(c)). In addition, the *C* allele generates a new binding site to transcription factor Nkx2.5 (YBYCACTTSM) that could favor *raet1e* gene expression only in positive cells for Nkx2.5 protein (Figure 1(d)). It would be necessary to conduct further experiments to corroborate this hypothesis.

On the other hand, rs6925151, rs9371533, rs2151910, and rs9383921 polymorphisms are located in coding regions and all of them produce amino acid changes. These changes could produce modifications in the raet1e protein, which may then have an effect in its linkage with the NKG2D receptor. In the mouse model, the lack of aortic expression of the *raet1e* gene results in increased atherosclerosis [9]. In humans, the participation of this molecule in the development of atherosclerosis has yet to be established; however, its participation in the immune process supports its role in this pathology. While in normal cells, raet1 proteins are either not expressed or expressed at low levels, these molecules are overexpressed under a stimulus, such as heat shock, DNA damage, or oxidative stress [33]. Oxidative stress is considered an important mechanism associated with the development of cardiovascular diseases [34, 35]. Several studies in human samples and animal models suggest that atherosclerotic plaques contain accumulated DNA damage and activated DNA damage response elements. In fact, it has been reported that the accumulation of DNA damage in atherosclerotic plaques is mediated, at least in part, by oxidative stress [36].

The activation of the NKG2D receptor in NK cells and some T cell subsets through raet1e stimulates the production of certain cytokines, such as interleukin 12, interferon-*γ*, and tumor necrosis factor-*α* (TNF-*α*). The important role of interferon-*γ* and TNF-*α* in the pathogenesis of atherosclerosis is well known [37]. It has been reported that IL-12, known as the Th1 response master controller, stimulates T and NK cells to produce IFN-*γ*, which induces multiple proatherogenic processes in the atherosclerotic lesion [2]. An important expression of both mRNA and protein of the IL-12 has been demonstrated in human atherosclerotic plaques [38], suggesting that plaque progression is supported by IL-12 production [39]. The possible role of the *raet1e* polymorphisms in either the protein expression levels or in the structural changes could in part regulate the raet1e molecule and thus modulate the different effects on all the immune phenomena related to the NKG2D receptor. Taken together, these facts could partially explain the association of these polymorphisms with CAD and with cardiovascular risk factors.

To the best of our knowledge, no studies have focused on the association of the *raet1e* polymorphisms with any disease. Interestingly, the gene is located in the q24.2–q25.3 region in chromosome 6 near the major histocompatibility complex (MHC) region. Genome-wide association (GWA) studies have revealed the correlation of MHC genes or intergenic regions in the three MHC classes of genes with cardiovascular diseases [40]. This region is one of the most polymorphic in humans and is well known for its role in the immunologic process.

One of the strengths of this study was the fact that our control group included only individuals without evidence of subclinical atherosclerosis defined using the coronary calcium score. In addition, population stratification was ruled out as a potential confounding factor, because the proportions of Caucasian, Native American, and African ancestries were similar in patients and healthy controls. Another strength of this study was that tomographic, clinical, and biochemical data were collected in both groups, which allowed us to adjust the analyses for a large number of potential confounding factors. Similarly, we demonstrated the functional effect of the rs7756850 polymorphisms using luciferase assays. Despite this, the limitations of this study should be considered. First, in this work, we have only included five polymorphisms of the *raet1e* gene, which seem to be functional and/or informative based on the analysis of the prediction software results. Second, conclusions on causality cannot be made because of the transversal character of the study. Third, since the selection of participants was not random, the findings may not be applicable to the general population; however, considering that the participants have no knowledge of their genotype, its distribution would be expected to be similar to a randomly selected sample. Finally, since the correlation of the *raet1e* polymorphisms with premature CAD and cardiovascular parameters is proved here for the first time, further studies in an independent group of patients are mandatory to validate the results.

## 5. Conclusions

In conclusion, this is the first study to report an association of the *raet1e* polymorphisms with an increased risk of developing premature CAD and with some cardiometabolic parameters. The functional effect of the rs7756850 was corroborated in luciferase assays.

## Figures and Tables

**Table 1:** Biochemical, clinical, demographic, lifestyle, and tomography characteristics of the study population.

	Control (*n* = 1104)	Premature CAD (*n* = 1158)	*p* value
Demographic			
Age (years)	51 ± 9	54 ± 8	<0.001
Gender (% male)	41.1	81.0	<0.001
Biochemical and clinical characteristics			
Body mass index (kg/m^2^)	27.9 [25.4-30.8]	28.3 [25.9-31.1]	0.004
Systolic blood pressure (mmHg)	112 [104-122]	116 [106-127]	<0.001
Diastolic blood pressure (mmHg)	71 [65-76]	71 [66-78]	0.011
Total cholesterol (mg/dL)	189 [166-210]	160 [132-193]	<0.001
High density lipoprotein cholesterol (mg/dL)	45 [36-55]	37 [32-44]	<0.001
Low density lipoprotein cholesterol (mg/dL)	115 [95-134]	91 [69-116]	<0.001
Triglycerides (mg/dL)	145 [107-201]	162 [119-219]	<0.001
Aspartate aminotransaminase (IU/L)	25 [21-30]	26 [22-31]	0.001
Gamma-glutamyltransferase (IU/L)	26 [18-42]	33 [22-49]	<0.001
Adiponectin (*μ*g/mL)	8.1 [5.0-12.8]	5.2 [3.2-8.1]	<0.001
Total cholesterol > 200 mg/dL (%)	36.6	30.2	<0.001
Hypo-*α*-lipoproteinemia (%)	52.0	67.2	<0.001
LDL pattern B (%)	47.0	62.0	<0.001
Low adiponectin (%)	42.8	57.5	<0.001
Hypertension (%)	18.9	68.0	<0.001
Type 2 diabetes mellitus (%)	9.8	35.6	<0.001
Increased aspartate aminotransaminase activity (%)	36.3	38.9	0.207
Increased gamma-glutamyltransferase activity (%)	41.3	49.9	<0.001
Tomography			
Total abdominal fat (cm^2^)	432 [347-534]	425 [339-523]	0.254
Visceral abdominal tissue (cm^2^)	139 [104-180]	168 [129-215]	<0.001
Subcutaneous abdominal fat (cm^2^)	286 [218-366]	245 [193-313]	<0.001
Lifestyle			
Current smoking (%)	22.8	11.7	<0.001

Data are shown as mean ± standard deviation, median [interquartile range], or percentage. Student *t* test, Mann-Whitney *U* test or chi-square test. CAD = coronary artery disease; HDL = high-density lipoprotein; LDL = low-density lipoprotein.

**Table 2:** Association of *Raet1e* gene polymorphisms with premature coronary artery disease.

Polymorphism	*n* (genotype frequency)			MAF	Model	OR [95% CI]	*p*
rs6925151	*C > T*						
	*CC*	*CT*	*TT*				
Control (*n* = 1104)	392 (0.355)	510 (0.462)	202 (0.183)	0.414	Heterozygote	1.250 [1.027-1.521]	0.026
CAD (*n* = 1158)	373 (0.322)	569 (0.491)	216 (0.187)	0.432	Codominant 1	1.268 [1.018-1.581]	0.034
rs9371533	*A > G*						
	*AA*	*AG*	*GG*				
Control (*n* = 1104)	293 (0.265)	523 (0.474)	288 (0.261)	0.498	Heterozygote	1.255 [1.031-1.527]	0.024
CAD (*n* = 1158)	314 (0.271)	585 (0.505)	259 (0.224)	0.476			
rs7756850	*G > C*						
	*GG*	*GC*	*CC*				
Control (*n* = 1104)	316 (0.285)	523 (0.474)	265 (0.240)	0.477	Heterozygote	1.274 [1.047-1.550]	0.016
CAD (*n* = 1158)	299 (0.258)	584 (0.504)	275 (0.237)	0.490	Codominant 1	1.294 [1.024-1.635]	0.031
rs9383921	*C > T*						
	*CC*	*CT*	*TT*				
Control (*n* = 1104)	318 (0.288)	525 (0.476)	261 (0.236)	0.474	Heterozygote	1.232 [1.012-1.499]	0.037
CAD (*n* = 1158)	318 (0.261)	584 (0.504)	272 (0.235)	0.487			

Data are expressed as odds ratio (OR) and 95% confidence interval (CI) as assessed by multivariate logistic regression analysis. Each model was adjusted for age, gender, body mass index, current smoking, and type 2 diabetes mellitus. MAF = minor allele frequency.

**Table 3:** Association of *raet1e* gene polymorphisms with clinical and metabolic parameters in the coronary patient group.

Polymorphism	*n* (genotype frequency)			MAF	Model	OR [95% CI]	*p*
Coronary patients							
Hypertension							
rs9371533	*AA*	*AG*	*GG*		Additive	1.264 [1.054-1.515]	0.012
No (*n* = 370)	113 (0.305)	188 (0.508)	69 (0.186)	0.441	Recessive	1.501 [1.092-2.063]	0.012
Yes (*n* = 788)	201 (0.255)	397 (0.504)	190 (0.241)	0.493	Codominant 2	1.629 [1.125-2.358]	0.010
Total cholesterol > 200 mg/dL							
rs9371533	*AA*	*AG*	*GG*				
No (*n* = 924)	261 (0.282)	448 (0.485)	215 (0.233)	0.475	Heterozygote	1.437 [1.071-1.926]	0.016
Yes (*n* = 234)	53 (0.226)	137 (0.585)	44 (0.188)	0.481			
rs2151910	*TT*	*TA*	*AA*				
No (*n* = 924)	426 (0.461)	391 (0.423)	107 (0.116)	0.327	Heterozygote	1.515 [1.133-2.027]	0.005
Yes (*n* = 234)	88 (0.376)	125 (0.534)	21 (0.090)	0.357	Codominant 1	1.508 [1.108-2.053]	0.009
GGT > p75							
rs7756850	*CC*	*CG*	*GG*				
No (*n* = 579)	166 (0.287)	273 (0.471)	140 (0.242)	0.478	Codominant 1	1.436 [1.080-1.909]	0.013
Yes (*n* = 579)	131 (0.227)	312 (0.538)	136 (0.234)	0.504			
rs9383921	*CC*	*CT*	*TT*				
No (*n* = 579)	168 (0.290)	273 (0.471)	138 (0.238)	0.474	Codominant 1	1.432 [1.078-1.903]	0.013
Yes (*n* = 579)	133 (0.229)	311 (0.538)	135 (0.233)	0.502			
Low adiponectin							
rs7756850	*CC*	*CG*	*GG*		Additive	1.237 [1.042-1.467]	0.015
No (*n* = 492)	137 (0.278)	257 (0.523)	98 (0.200)	0.460	Recessive	1.493 [1.119-1.991]	0.006
Yes (*n* = 666)	163 (0.244)	324 (0.487)	179 (0.269)	0.512	Codominant 2	1.538 [1.096-2.185]	0.013
rs9383921	*CC*	*CT*	*TT*		Additive	1.257 [1.059-1.490]	0.009
No (*n* = 492)	140 (0.284)	256 (0.521)	96 (0.195)	0.455	Recessive	1.509 [1.129-2.016]	0.005
Yes (*n* = 666)	161 (0.242)	326 (0.490)	179 (0.268)	0.514	Codominant 2	1.600 [1.132-2.260]	0.008

Data are expressed as odds ratio (OR) and 95% confidence interval (CI) as assessed by multivariate logistic regression analyses. All models were adjusted by age, gender, and body mass index. MAF = minor allele frequency; GGT = gamma-glutamyltransferase.

**Table 4:** Association of *raet1e* gene polymorphisms with clinical and metabolic parameters in the control group.

Polymorphism	*n* (genotype frequency)			MAF	Model	OR [95% CI]	*p*
Controls							
Hipo-*α*-lipoproteinemia							
rs9371533	*AA*	*AG*	*GG*				
No (*n* = 530)	125 (0.236)	277 (0.522)	128 (0.242)	0.503	Heterozygote	0.694 [0.545-0.885]	0.003
Yes (*n* = 574)	168 (0.293)	246 (0.429)	160 (0.279)	0.493	Codominant 1	0.658 [0.490-0.884]	0.005
LDL pattern B							
rs566850	*CC*	*CG*	*GG*				
No (*n* = 585)	153 (0.262)	277 (0.473)	154 (0.265)	0.502	Additive	0.815 [0.690-0.964]	0.017
Yes (*n* = 519)	164 (0.316)	245 (0.473)	110 (0.211)	0.448			
rs9383921	*CC*	*CT*	*TT*				
No (*n* = 585)	158 (0.270)	273 (0.466)	154 (0.263)	0.496	Recessive	0.710 [0.533-0.946]	0.019
Yes (*n* = 519)	161 (0.310)	251 (0.484)	107 (0.206)	0.409			
AST > p75							
rs7756850	*CC*	*CG*	*GG*				
No (*n* = 705)	184 (0.261)	343 (0.486)	178 (0.253)	0.496	Dominant	0.720 [0.549-0.944]	0.018
Yes (*n* = 399)	133 (0.333)	179 (0.446)	88 (0.221)	0.387			
rs9383921	*CC*	*CT*	*TT*				
No (*n* = 705)	183 (0.260)	345 (0.489)	177 (0.251)	0.496	Additive	0.800 [0.673-0.951]	0.011
Yes (*n* = 399)	136 (0.341)	178 (0.446)	85 (80.213)	0.436	Dominant	0.695 [0.695-0.911]	0.008
					Codominant 2	0.651 [0.461-0.919]	0.015

Data are expressed as odds ratio (OR) and 95% confidence interval (CI) as assessed by multivariate logistic regression analyses. All models were adjusted by age, gender, and body mass index. MAF = minor allele frequency; AST = aspartate aminotransaminase.

## Data Availability

No data were used to support this study.
